# [Corrigendum] Upregulation of H19 promotes invasion and induces epithelial-to-mesenchymal transition in esophageal cancer

**DOI:** 10.3892/ol.2026.15879

**Published:** 2026-09-23

**Authors:** Cheng Huang, Lihua Cao, Limin Qiu, Xiaoli Dai, Linwei Ma, Yingting Zhou, Huifen Li, Min Gao, Weiyong Li, Qing Zhang, Koulan Han, Hongzhen Lv

Oncol Lett 10: 291–296, 2015; DOI: 10.3892/ol.2015.3165

Following the publication of this paper, it was drawn to the Editor's attention by a concerned reader that, regarding the cell invasion assay data shown in Fig. 2C and D on p. 294, the ‘pcDNA’ panel in Fig. 2C and the ‘siRNA/H19’ panel in Fig. 2D contained an overlapping section of data, suggesting that these data had been derived from the same original source, even though the results from differently performed experiments were intended to have been portrayed. In addition, the nucleotide sequence featured in the Materials and methods section that was reported to target GAPDH with the sequence 5’-CTGTCCTCGCCGTCACACCG-3’ was instead predicted to target H19/LRP8 according to a BLAST search, suggesting that the sequences for certain of the primers may have been written incorrectly in the paper. Finally, an independent analysis of the data in this paper undertaken by the Editorial Office revealed that data featured in Fig. 2C were strikingly similar to data that subsequently appeared in a paper featuring an author of the same name (Huifen Li, although the research institutes were shown to be different), which was published in the journal *Diagnostic Pathology*, although that article has since been retracted.

The authors have replied to the Editorial Office to confirm that the sequence of the GAPDH primer was inadvertently written incorrectly in the Materials and methods section; the primers in question for GAPDH should have been written as follows:

Forward: 5’-TGCACCACCAACTGCTTAGC-3’

Reverse: 5’-GGCATGGACTGTGGTCATGAG-3’.

Concerning the issue of the overlapping data panels in Fig. 2C and D, the authors declared that they no longer had access to their original data, however, they were able to repeat the affected cell invasion assay experiments, and a new version of Fig. 2, showing the revised data for Fig. 2C and D, is shown on the next page. The authors are able to confirm that the new results are in broad agreement with those obtained originally, and that the results and the conclusions of this study have not been significantly altered by replacing these data. The replacement of the original data with the new data also attends to the issue of the apparent reappearance of the former data in the journal *Diagnostic Pathology*.

The authors are grateful to the Editor of *Oncology Letters* for allowing them the opportunity to publish this Corrigendum, and all the authors agree with its publication. They also thank the reader of the article for drawing these matters to their attention.

## Figures and Tables

**Figure 2.**
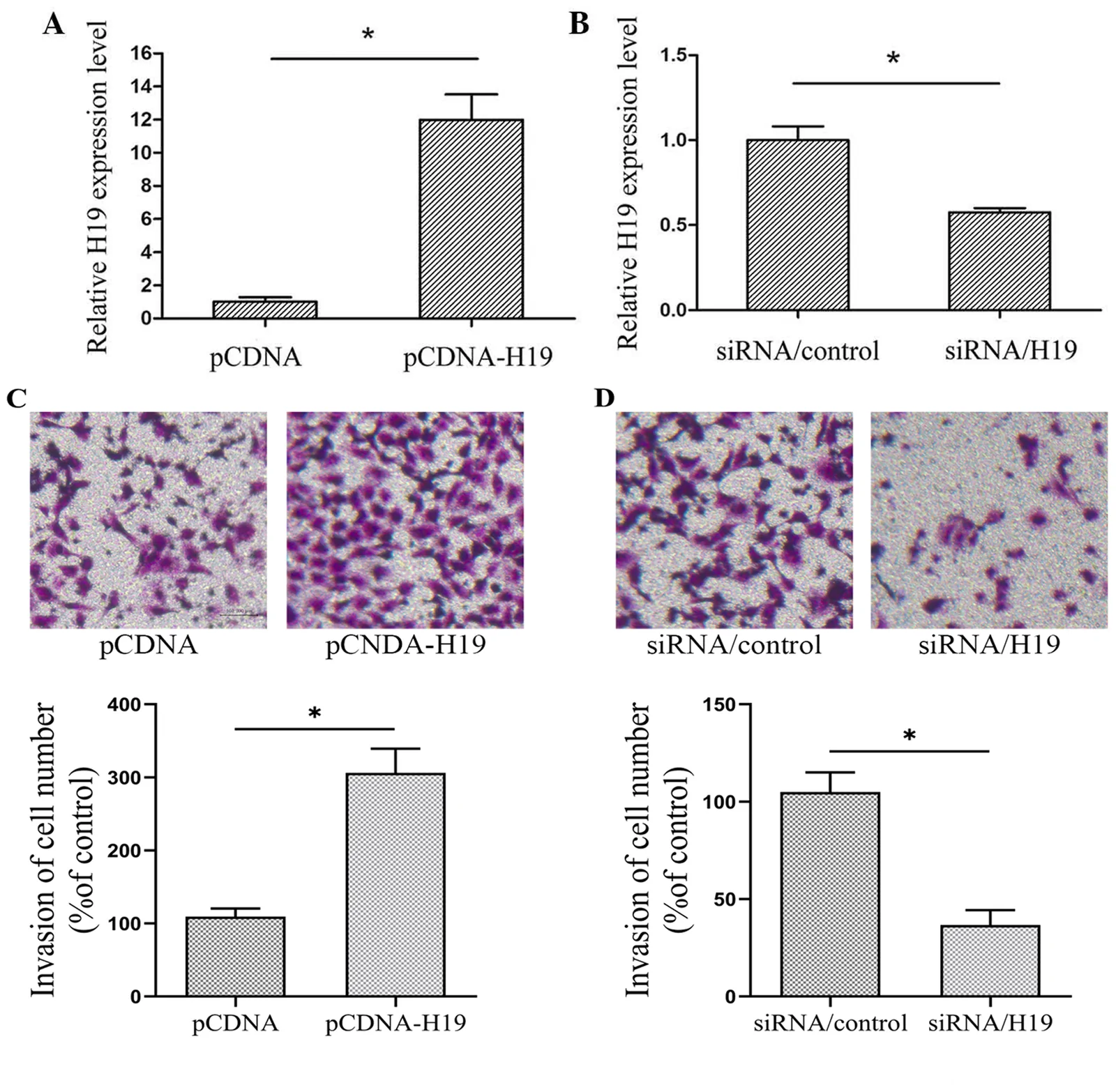
H19 regulates the invasive ability of cells. The expression of H19 in Eca-109 cells transfected with (A) pCDNA or pCDNA-H19 and (B) siRNA/control or siRNA/H19. The transfection efficiency was validated by RT-qPCR. A Transwell assay was performed in order to determine to invasive ability of cells treated with (C) pCDNA or pCDNA-H19 (magnification, ×20) and (D) siRNA/control or siRNA/H19 (magnification, ×20) for 24 h. The representative images reveal invasive cells on the lower side of the membrane stained with crystal violet. The quantifications of cell invasion are presented as a percentage of the control cell number. All experiments were performed in triplicate and are presented as the mean ± standard error of the mean. *P<0.05. Each independent experiment was performed three times. siRNA, small interfering RNA.

